# Combination Therapy With Voclosporin and Anifrolumab in Two Patients With Lupus Nephritis and Discoid Lupus Erythematosus

**DOI:** 10.7759/cureus.83337

**Published:** 2025-05-02

**Authors:** Arbor G Dykema, Ziga Vodusek, Ralina Karagenova, Adam Krieger, Homa Timlin

**Affiliations:** 1 Dermatology, Johns Hopkins University School of Medicine, Baltimore, USA; 2 Rheumatology, Johns Hopkins University School of Medicine, Baltimore, USA; 3 Rheumatology, University of Hawai'i, John A. Burns School of Medicine, Honolulu, USA

**Keywords:** anifrolumab, discoid lupus, lupus nephritis, systemic lupus erythematosus, voclosporin

## Abstract

Current therapeutic options in systemic lupus erythematosus (SLE) vary in effectiveness, and remission may not be achievable in many patients. Unfortunately, although many patients achieve an initial response with conventional immunosuppressant regimens, relapse is common, and current therapies are associated with damaging adverse effects and the risk of infection. Therefore, it is critical to identify safe targets for lupus disease activity, minimizing drug toxicity and preventing damage accrual. We present two cases with disease features of discoid lupus erythematosus (DLE) and lupus nephritis (LN), who were treated safely and successfully with Voclosporin and Anifrolumab, resulting in disease remission without the use of oral steroids.

## Introduction

The benefits of treat-to-target (T2T) have been demonstrated in chronic diseases [1]. The arrival of novel therapies will enable the routine implementation of T2T approaches in patients with systemic lupus erythematosus (SLE). Anifrolumab was Food and Drug Administration (FDA) approved in 2021 and European Medicines Agency (EMA) approved in 2022 for the treatment of adults with SLE [2], and Voclosporin was also FDA approved in 2021 and EMA approved in 2022 for patients with SLE and lupus nephritis (LN) [3]. We present two cases of patients with LN and discoid lupus erythematosus (DLE) who were successfully treated with a combination of Anifrolumab and Voclosporin, allowing for the weaning and eventual cessation of long-term steroids. The first case involves a patient with LN and refractory DLE who showed significant clinical improvement within six months of initiating dual therapy. The second case describes a patient who initially responded well to combination therapy but experienced relapse after discontinuation. Resumption of treatment led to disease control, highlighting the long-term safety of this regimen and the effectiveness of Anifrolumab in managing cutaneous disease in an older patient.

## Case presentation

Case 1

**Figure 1:**
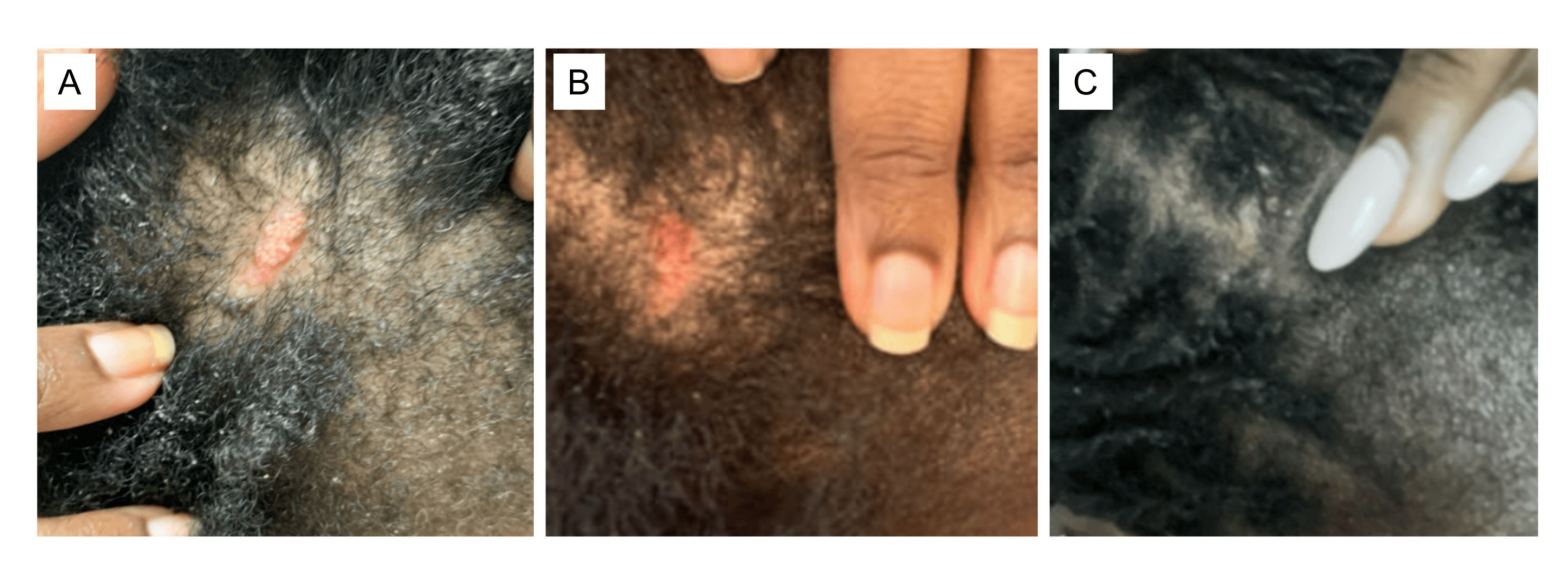
Patient 1 with a DLE lesion prior to starting Anifrolumab and rapid resolution of active disease after therapy initiation DLE lesion on the scalp two weeks prior to (A) and after three (B) and five months (C) of Anifrolumab therapy. DLE: discoid lupus erythematosus

A 36-year-old African American woman was diagnosed with systemic lupus erythematosus (SLE) in her early 20s, characterized by positive antinuclear antibody (ANA), anti-double-stranded DNA (anti-dsDNA), anti-Smith, arthritis, hemolytic anemia, leukopenia, thrombocytopenia, pleurisy, mucosal ulcers, discoid lupus erythematosus (DLE), alopecia, and lupus nephritis. Her treatment history included variable courses of steroids, hydroxychloroquine, mycophenolate mofetil, methotrexate, azathioprine, and belimumab. Despite these modalities, her disease remained active with frequent arthritis, mucosal ulcers, DLE, and proteinuria (peak of 800 mg/24 hours). In 2017, a kidney biopsy confirmed class III/V lupus nephritis. Arthritis resolved when she was treated with a combination of rituximab and belimumab [4], and proteinuria resolved after initiating Voclosporin in 2021 (15.8 mg twice daily). A repeat kidney biopsy in October 2024 demonstrated lupus nephritis class II. DLE and alopecia remained active, requiring intralesional triamcinolone injections every one to two months (Figure 1A). One month after her kidney biopsy, Anifrolumab was initiated, resulting in significant improvement of her DLE rash by three months (Figure 1B), and almost complete resolution by six months of treatment (Figure 1C). Since then, she has successfully tapered off oral prednisone and has not required intralesional triamcinolone. In summary, a patient with refractory SLE who had failed multiple conventional and immunosuppressive therapies experienced marked clinical improvement in both her DLE and LN after the initiation of Voclosporin and the subsequent addition of Anifrolumab. She was able to discontinue steroids and intralesional injections and has remained stable on dual therapy without reported infections or notable side effects.

Case 2

**Figure 2:**
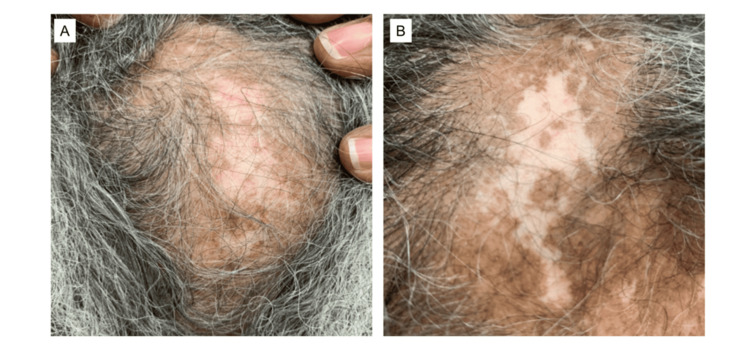
Patient 2 with active scalp DLE prior to starting Anifrolumab and resolution of active disease after three months of therapy Active DLE lesion on the scalp prior to Anifrolumab therapy (A). Improved rash after three months of Anifrolumab therapy (B). DLE: discoid lupus erythematosus

A 70-year-old African American woman was diagnosed with SLE 40 years ago, distinguished by positive ANA, dsDNA, and Smith antibodies, as well as systemic symptoms such as DLE, arthritis, mucosal ulcers, pleurisy, and alopecia. Due to frequent flares of arthritis and DLE, she trialed multiple medications, including azathioprine, mycophenolate mofetil, and methotrexate in addition to chronic hydroxychloroquine and steroids. Her arthritis improved on baricitinib; however, her scalp DLE remained active (Figure 2A), requiring frequent intralesional triamcinolone injections to the scalp. Later in her disease course, she developed proteinuria with a peak of 7 g. A kidney biopsy at this time confirmed lupus nephritis class V. After transitioning from baricitinib to Voclosporin (23.7 mg twice daily), her proteinuria normalized within 6 months. Subsequently, Anifrolumab was added with the hopes of achieving better control of her cutaneous disease. The patient demonstrated significant improvement in her DLE by the third month of Anifrolumab infusions (Figure 2B) and was able to taper off oral prednisone, intralesional triamcinolone injections, as well as mycophenolate. She has not had further flares of arthritis or proteinuria. Her course was complicated by a urinary tract infection for which she received oral antibiotics and resolved without complications. We followed her for 25 months, and her disease remained stable on combination therapy.

**Figure 3:**
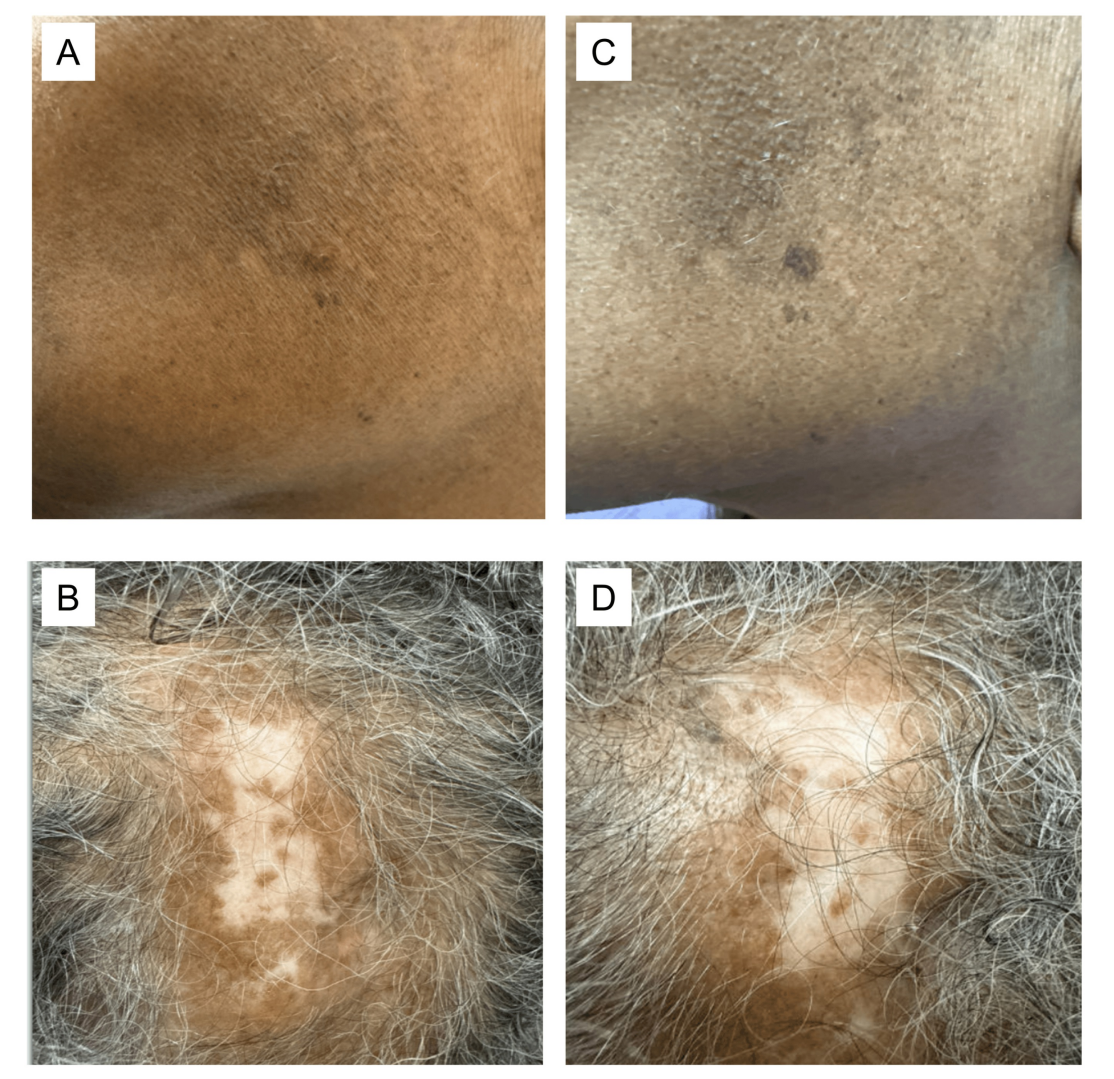
Relapse of disease while off Anifrolumab with rapid resolution of lesions after restarting therapy New (A) and active, erythematous (B) lesions while off Anifrolumab for six months. Complete resolution of active disease two weeks after restarting Anifrolumab infusions with residual mild hyperpigmentation (C, D).

She was taken off Anifrolumab following a fall and scalp fracture that required neurosurgical intervention. Voclosporin was held briefly postoperatively, then resumed at a reduced dose of 15.9 mg twice daily. Subsequently, the patient developed new skin lesions while being off Anifrolumab for six months (Figures 3A, 3B). Briefly, her urine protein-to-creatinine ratio (UPCR) had risen to 0.38 gr/g creatinine (normal range: 0.00-0.19). The remaining labs revealed persistently positive dsDNA and a new onset of hypocomplementemia. Her skin lesions resolved within two weeks of resuming Anifrolumab (Figures 3C, 3D). During this period, the patient did not experience any new infections.

In summary, this elderly patient demonstrated a response to both renal and cutaneous disease, with successful tapering off corticosteroids and mycophenolate. Notably, she tolerated combination therapy well, without any major infections or treatment-related side effects, emphasizing the safety of this regimen even in older individuals who may be at higher risk for complications. Her disease flared during a temporary interruption of both Voclosporin and Anifrolumab, particularly in the skin, highlighting her high risk for flares. Upon reintroduction of Anifrolumab, her skin lesions resolved, suggesting its key role in maintaining disease control.

## Discussion

Severe DLE can be refractory to traditional immunosuppressants, and LN significantly impacts the overall prognosis of patients with SLE, making these disease presentations a difficult and essential treatment target in SLE [5,6]. Moreover, prolonged glucocorticoid use is associated with irreversible organ damage [7]. These two cases provide emerging evidence supporting the efficacy and safety of combination therapy with Voclosporin and Anifrolumab in patients with SLE presenting with both LN and DLE; with Voclosporin improving LN and Anifrolumab predominantly having activity against cutaneous disease (DLE). Here, we build on previous findings by offering longer-term follow-up and introducing a second case, further reinforcing the therapeutic potential of this approach. Importantly, both patients were able to maintain a clinical response without the use of steroids, an essential goal in the long-term management of SLE.

## Conclusions

Together, these cases provide early evidence suggesting the potential effectiveness of combining Voclosporin and Anifrolumab while also allowing the tapering of mycophenolate and steroids. However, the study is limited by its small sample size. Further research, ideally randomized controlled clinical trials, is warranted to better define the utility and effectiveness of this combination therapy and to determine whether early initiation could replace mycophenolate and steroids in the treatment of DLE and LN.
